# Intercultural sensitivity as a mediator in the relationship between implicit intercultural identification and emotional disturbance—An exploratory study of international high school students

**DOI:** 10.3389/fpsyt.2023.1098671

**Published:** 2023-04-28

**Authors:** Jiayin He, Xiaoqi Song, Chanyu Wang, Ruibin Zhang

**Affiliations:** ^1^Laboratory of Cognitive Control and Brain Healthy, Department of Psychology, School of Public Health, Southern Medical University, Guangzhou, China; ^2^Department of Psychiatry, Zhujiang Hospital, Southern Medical University, Guangzhou, China

**Keywords:** intercultural communication competence, implicit intercultural identification, SC-IAT, emotional disturbance, intercultural sensitivity

## Abstract

**Background:**

Intercultural adaptation is always associated with emotional disturbances. Intercultural communication competence, as an important competence of intercultural adaptation, involves implicit intercultural identification and intercultural sensitivity. Competence in these areas promotes the process of intercultural adaptation. Little is known about the relationship between intercultural communication competence and emotional disturbances in new students attending international high schools. The intercultural adaptation process of this population requires attention because of the increasing number of high school students attending international schools, and the fact that most of these teenagers are immersed in intercultural contexts for the first time.

**Objective:**

This study aimed to investigate the prevalence of emotional disturbance in new students in an international high school and confirm the relationship among implicit intercultural identification, intercultural sensitivity, and emotional disturbances.

**Methods:**

Study 1 was an investigation of the prevalence of emotional disturbance in 105 students in their 1st year at an international high school using the Self-rating Depression Scale and Self-rating Anxiety Scale. Of these students, 34 were invited to participate in Study 2 to further explore the relationship between intercultural sensitivity, implicit intercultural identification, and emotional disturbances using the Intercultural Sensitivity Scale and Single Category Implicit Association procedure.

**Results:**

Study 1 indicated that 15.24% of students were affected by apparent depression and 10.48% had anxiety symptoms. Study 2 revealed that emotional disturbances significantly correlated with intercultural sensitivity (*p* < 0.01) and implicit intercultural identification (*p* < 0.01). The openness factor from intercultural sensitivity mediated the relationship between implicit intercultural identification and depression (ratio of indirect effect = 41.04%, *p* < 0.05) and anxiety symptoms (ratio of indirect effect = 34.65%, *p* < 0.05).

**Conclusion:**

The study revealed that a significant proportion of students in the 1st year of international high school are affected by emotional difficulties. However, intercultural communication competence is a protective factor. Enhancing the international communication competence of senior students in international high schools is important to mitigate mental health challenges.

## Introduction

The economic and political exchange between countries increases the demand for intercultural talent, and international schools—bridges to intercultural globalization—are becoming increasingly popular. Typically established in major cities around the world, the number of students attending international schools reached approximately 6 million in 2020 (1). Studies indicate that students learning in intercultural contexts will be more likely to experience emotional disturbances, such as depression and anxiety, than students in a single culture because of the arduous process of intercultural adaptation (2, 3). The different aspects of intercultural communication competence are instrumental to understanding intercultural adaptation (4). Numerous studies have focused on foreign students in college, while a smaller number have targeted those in international schools, especially students entering their 1st year of international high school. Most students starting at an international high school have received monocultural elementary education before going to an international school and must face the sudden change to an intercultural environment. Will they be emotionally affected during the process of intercultural adaptation? Do they have intercultural communication competence? Can intercultural communication competence help them manage their emotional challenges? Addressing these questions is the focus of this article.

### Intercultural adaptation of students in international high schools

International schools were first set up, a century ago, to provide education for children of expatriate workers (5). In 2000, there were 2,584 English international schools worldwide, and most of the students attending these schools were non-natives of the country in which the schools were based. In 2021, the number of schools exceeded 11,000, with 80% of students attending them being considered natives (1). To successfully enter prestigious universities in Western countries, many students attend international high schools, which offer bridging curricula linked to Western university admission applications.

When students enter international high school, they must go through the process of intercultural adaptation. Research suggests that individuals living in an intercultural environment face a higher risk of mental health difficulties than those living in their local culture (6). Nguyen and his colleagues reported that 37.81% of foreign students who attended the Japanese university were affected by depression, while the proportion of domestic students affected was 29.85% (2019). Individuals often immerse themselves in the new environment, idealizing the intercultural experience at the outset of intercultural adaptation. Yet differences—big or small—between the new setting and the previous familiar environment lead rapidly to many intercultural challenges in daily life (4). Intercultural adaptation can be difficult and can affect one's mental state because long-held habits and communication patterns need to be adjusted for the new culture (7–9).

Compared with individuals studying in foreign countries, it seems that domestic students in international schools experience a milder transition. They live in an intercultural environment dominated by their own culture. Nevertheless, research on non-local students in Taiwan—including foreign students, students from China, Hong Kong, Macau, and overseas Chinese students—reported that 40.9% of students suffered from mild-to-extreme depression, and 48.6% suffered from mild-to-extreme anxiety. Hong Kong and Macao students had the highest levels of depression, anxiety, and stress (10). Even though many of these students had grown up in the context of Chinese culture, the intercultural environment still had a negative effect on their mental health. Most students receive traditional domestic education before they study in international high schools. The sudden change in cultural context may lead to unpredictable emotional responses. Furthermore, younger students are more likely to be emotionally affected because adolescents typically experience more frequent high-intensity emotions than adults (11).

### Intercultural adaptation and mental disorders

Emotional disturbances, such as depression and anxiety, are the most common mental health difficulties encountered during the challenging process of intercultural adaptation (3). Capps et al. (12) found that more than half of the Latino students in an American international high school reported being disturbed by negative emotions: two-thirds met the clinical threshold for anxiety, and 55% met the depression threshold. Anxiety is often associated with intercultural communication, and the Anxiety/Uncertainty Management (AUM) theory suggests that anxiety is related to the uncertainty of intercultural communication, supported by research with Japanese and US college students (13). Anxiety has also been noted as a significant predictor of willingness for intercultural interaction (14, 15). A lack of intercultural knowledge will lead to anxiety when individuals are exposed to a new culture. To avoid anxiety, individuals may reject intercultural communication, resulting in serious obstructions to intercultural adaptation in turn. Moreover, depression is related to a decline in life quality caused by intercultural dilemmas. Research involving 225 Vietnamese students enrolled in a language course at a college in Busan found that depression correlated with life dissatisfaction during the process of intercultural adaptation (16). Han and colleagues surveyed 130 Chinese international students at Yale University in the US and found that those who reported depressive symptoms identified culture shock as an important cause of mental distress (17). In summary, it is imperative to concentrate on the intercultural adaptation of students in international high schools by investigating their depression and anxiety levels after enrollment. Important competences that can promote the process of intercultural adaptation are discussed in the following paragraph.

### Crux of intercultural adaptation: intercultural communication competence

Intercultural communication competence, defined as an ability to interact effectively and appropriately with people and the environment in an intercultural context, is regarded as an important competence needed for intercultural adaptation. Intercultural communication competence emphasizes how to fulfill one's own communication goals through respect, tolerance, and integration of cultural differences (4, 18, 19). Intercultural communication competence can be viewed from different aspects.

#### Implicit intercultural identification

As the cognitive aspect of intercultural communication competence, intercultural awareness is a process of attitudinal internalization of understanding and appreciating cultural differences in intercultural communication. Intercultural communication consists of three levels: (1) awareness of cultural traits, (2) learning the significant and subtle differences through comparison from the experience of different cultures, and (3) learning and identifying another culture from the insider's perspective autonomously (4). Intercultural identification is the highest level of intercultural awareness, and individuals who attain this level can appreciate cultural differences and enjoy intercultural life.

Most studies have focused on the evaluation of cultural traits and differences (20, 21), while fewer have concentrated on intercultural identification. The promotion of cultural globalization means that teenagers can obtain a large amount of intercultural information through traveling, browsing websites, or making intercultural friends online, leading readily to the formation of intercultural identification (22, 23). Studies conducted in intercultural contexts have indicated that some teenagers identify rapidly with the local culture upon entering the intercultural environment. They can combine it with their own culture, and a few even acquire a bicultural identity (24–26).

It is suggested that intercultural identification can help individuals maintain better mental health in intercultural contexts and those who reject to identify with the new culture have a higher risk of emotional disturbance (27, 28). Research on adolescents indicated that intercultural identification plays a more notable role than adults in the process of intercultural adaptation (29). Adolescents are more easily affected by social requirements and conflicts because their stage of development is critical for self-identity construction (30). Students in intercultural environments may find it more difficult to construct their self-identity than students in ordinary schools because of intercultural conflicts caused by the immense value differences. The identity confusion may, in turn, hinder them from intercultural adaptation and lead to serious emotional disturbance (30, 31).

Additionally, self-report measures are often used in studies of intercultural identification (32, 33). A meta-analysis revealed that self-report measures of explicit attitudes are readily affected by social expectations and a lack of validity in some cases (34, 35). Implicit attitudes remain more stable over time, often unconsciously affecting people's words and deeds (36). In other words, implicit intercultural identification is more significant for the process of intercultural adaptation and is considered an important predictor of intercultural cognition (37). Based on these findings, we investigated the level of implicit intercultural identification of students in an international high school and explored whether this was related to emotional disturbances in the process of intercultural adaptation.

#### Intercultural sensitivity

Intercultural sensitivity, the ability to project and receive positive emotions for the sake of appropriate and effective intercultural interaction, is considered the affective aspect of intercultural communication competence (38). Self-esteem, self-monitoring, open-mindedness, empathy, interaction involvement, and non-judgment are essential qualities of intercultural sensitivity (4). Some researchers emphasize that openness is a key to intercultural sensitivity. It can improve intercultural attitudes and reduce intercultural prejudice (39, 40). Open-minded individuals accept cultural discrepancies quickly and can immerse themselves in unfamiliar intercultural environments.

Intercultural sensitivity is a key to intercultural adaptation (41). Individuals with high levels of intercultural sensitivity can better perceive and express positive emotions in the process of intercultural communication and are less disturbed by negative emotions (14, 38, 42). Importantly, positive emotions can help individuals overcome cultural differences in intercultural contexts (4). A quantitative study of 300 international students in an undergraduate program reported that intercultural sensitivity was a predictor of intercultural life satisfaction (43). In addition, the emotional distress of international high school students in intercultural environments may be more closely related to intercultural sensitivity than adults because emotions and feelings are complex and unstable in adolescence (44). Thus, the investigation of international high school students' intercultural sensitivity, and the relationship between intercultural sensitivity and emotional disturbances during intercultural adaptation, is important.

#### Relationship between implicit intercultural identification and intercultural sensitivity

Implicit intercultural identification and intercultural sensitivity are two important components of intercultural communication competence that represent the cognitive and affective aspects, respectively. Theories from the cognitive aspect consider that learning about a new culture is the basis for individuals' acquisition of intercultural communication competence—assuming effective intercultural interaction and promoting positive feelings toward the search for intercultural coexistence (19, 38). Mixed method research also revealed that intercultural awareness impacts intercultural sensitivity (45). However, less research has explored the relationship between implicit intercultural identification and intercultural sensitivity in detail.

### Current investigation

**Figure 1:**
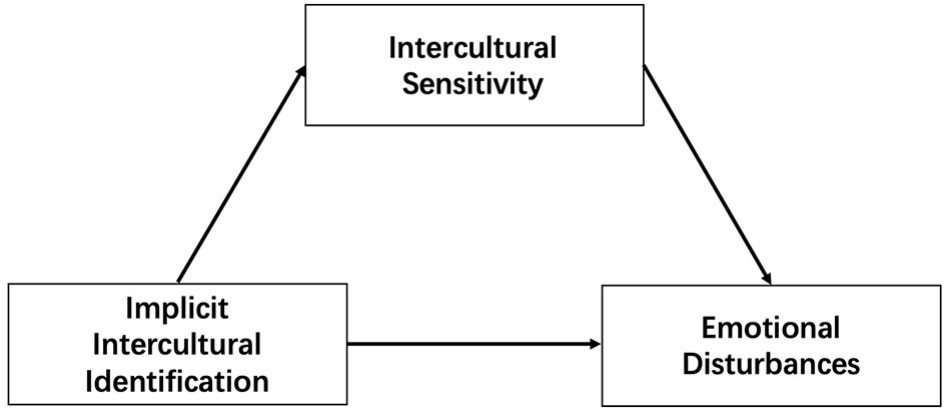
Proposed model for the relationship among implicit intercultural identification, intercultural sensitivity, and emotional disturbances.

This study aimed to investigate the proportion of students with symptoms of depression and anxiety in their 1st year (senior one) of international high school, linking their mental health to multiple core elements of intercultural adaptation. We set out to delineate the important role of intercultural adaptation in mental health and extend previous studies. Study 1 focused on the prevalence of senior-one students' emotional disturbances. Study 2 further explored the relationship among intercultural sensitivity, implicit intercultural identification (representing two aspects of intercultural communication competence), and emotional disturbances. We hypothesized that senior-one students in international high schools experience symptoms of depression and anxiety (Hypothesis 1), especially those with low intercultural sensitivity and implicit intercultural identification (Hypothesis 2). In addition, the theoretical assumptions of the cognitive and affective aspects of intercultural communication competence suggest that implicit intercultural identification could affect students' emotional states through intercultural sensitivity (Hypothesis 3), as indicated in the model shown in Figure 1.

## Study 1

### Study design

Study 1 investigated the prevalence of depression and anxiety in students in their 1st year of international high school. We set out to verify Hypothesis 1 in Study 1.

### Sample and procedure

All participants were senior-one students in an international school. This school is located in a metropolis of South China, with courses of Advanced Placement authorized and approved by the US College Board. Data were collected 1 month after students' admission. Informed consent was obtained from students and their parents, and the students could refuse to participate at any time. From a total cohort of 119 senior-one students, 105 students agreed to participate in the study. Of these students, 54 were male patients (aged 15 ± 0.42) and 96% of the sample was local. The participants were asked to complete two questionnaires related to emotional difficulties using the online platform (PsyEdu, https://xl.psyedu.cn/gdify/login) within a 2-day period.

### Measurement tools

We used the Self-rating Depression and Anxiety Scales to evaluate the emotional status of students.

#### Self-rating depression scale

The SDS is a short self-rating scale to assess the psychological and somatic symptoms of depression (46). The correlation coefficient between the SDS and psychiatric diagnosis was 0.69 (47), and the factor analysis indicates that the SDS has a good construct validity for Chinese adolescents (48). The Cronbach's α in this study was 0.818. The SDS contains 20 items based on the diagnostic criteria for depression. Participants rate each item on a 4-point Likert scale regarding how they have felt during the past weeks. The raw sum scores of the SDS range from 20 to 80. Raw scores are converted to index scores, and index scores exceeding 53 indicate apparent depression (46).

#### Self-rating anxiety scale

The SAS was developed to assess the frequency of anxiety symptoms based on diagnostic conceptualizations. It has been widely used in research and clinical practice (49). Research with Chinese middle school students indicated that the split-half reliability of SAS was 0.696, and the test–retest reliability at an interval of 1 month was 0.777 (50). The Cronbach's α was 0.822 in this study. There are 20 items in the SAS, and the respondent indicates how often they have experienced each symptom. The SAS scoring rules are similar to those of the SDS with the index score's standard line of apparent anxiety at 50 (49).

### Statistical analysis

Data were analyzed using SPSS version 21.0 (IBM Corp.: Armonk, NY) and SPSSAU. Descriptive statistics were used to summarize anxiety and depression prevalence. *T*-tests were used to compare gender differences, and analysis of variance (ANOVA) was used to compare age differences.

### Results

With 53 points as the critical value of the SDS (*mean* = 43.56, *SD* = 8.82) and 50 points as the critical value of the SAS (*mean* = 39.25, *SD* = 8.46), 16 students were considered to have apparent depressive symptoms (15.24%). A total of 11 students had apparent anxiety (10.48%). No gender and age differences were found in the occurrence of anxiety and depression (*p* > 0.05). Table 1 presents the demographic characteristics of students discriminating between apparent anxiety and depression.

**Table 1:** Demographic characteristics of students discriminating on apparent anxiety/depression (*N* = 105).

**Scale**	**Variables**	**Score > critical value**	***t* / F**	***p***
**SDS**	**Sex**			
	Male	9 (16.67%)	−0.42	0.68
	Female	7 (13.73%)		
	**Age**			
	14	1 (11.11%)	0.99	0.37
	15	15 (17.24%)		
	16	0 (0.00%)		
**SAS**	**Sex**			
	Male	5 (9.26%)	0.42	0.68
	Female	6 (11.76%)		
	**Age**			
	14	1 (11.11%)	0.01	1.00
	15	9 (10.34%)		
	16	1 (11.11%)		

SDS, Self-rating Depression Scale; SAS, Self-rating Anxiety Scale.

### Discussion

The results of Study 1 revealed that approximately 10% of senior-one students in an international high school were emotionally affected by depression and anxiety. This is a relatively lower rate than for non-local students for whom the prevalence was reported at one-third of the population (10, 51). The proportion of anxiety/depressive symptoms in the current study was also lower than that reported in the only similar recent research carried out in an international high school, which focused on immigrant Latino students in the US (12). Therefore, our results confirm that compared with individuals studying in foreign countries, domestic students in international schools experience a milder adaptive process.

Differences in the proportion of affected students may be caused by the nature of the participants. Most participants in previous studies were non-local students, while the majority of students in the current study were local. Although both groups experience the process of intercultural adaptation, local students do not leave the culturally familiar environment, while non-local students are immersed in environments that are new to them. Foreign students encounter more stressors, the impact of immigration, and greater culture shock (12) meaning the challenges of intercultural adaptation are greater for them.

Furthermore, Chen (4) summarized theories about the process of intercultural adaptation and concluded that individuals will idealize their lives in the early stages of intercultural adaptation. The idealization has a positive psychological effect. While the difficulties from reality that follow will end the idealization and result in mental distress. All participants in the current study were senior-one students who were new to an intercultural environment. Thus, most were not affected by anxiety and depression immediately after admission. Follow-up studies are needed in the future.

## Study 2

### Study design

Study 1 revealed the prevalence of mental health challenges faced by senior-one students in international high schools. Study 2 was conducted to further explore whether intercultural communication competence was protective against depression and anxiety symptoms, and how different aspects of intercultural communication competence work in an intercultural context.

### Sample and procedure

Students were asked whether they would like to participate in Study 2 after the completion of Study 1. Informed consent was obtained before the study began. In total, 34 students (28 girls, aged 14.94±0.34, all of them were local) participated in Study 2. They were asked to complete a questionnaire about intercultural sensitivity and participate in an experiment on implicit intercultural identification individually within 1 week. The experimental environment (including the instructor, instruction, and computer) was the same for each participant to reduce the influence of unrelated variables.

### Measurement tools

#### Implicit intercultural identification

We focused on implicit intercultural identification, which represents the third level of intercultural awareness. Single Category Implicit Association (SC-IAT) designed by E-prime (version 1.1) was used to measure implicit cognition. SC-IAT shows the strength of evaluative associations with a single attitude object (52) and has reasonable internal consistency in practical applications. Its reliability coefficient ranges from 0.55 to 0.85, and the average reliability coefficient is 0.69 (52, 53).

The materials of SC-IAT require the collection of 10 self/other attribute words (e.g., myself/other). We also selected seven words that students (excluding those in the same international school attended by the study participants) believed the best to represent Western culture (e.g., Hollywood), using a questionnaire.

**Figure 2:**
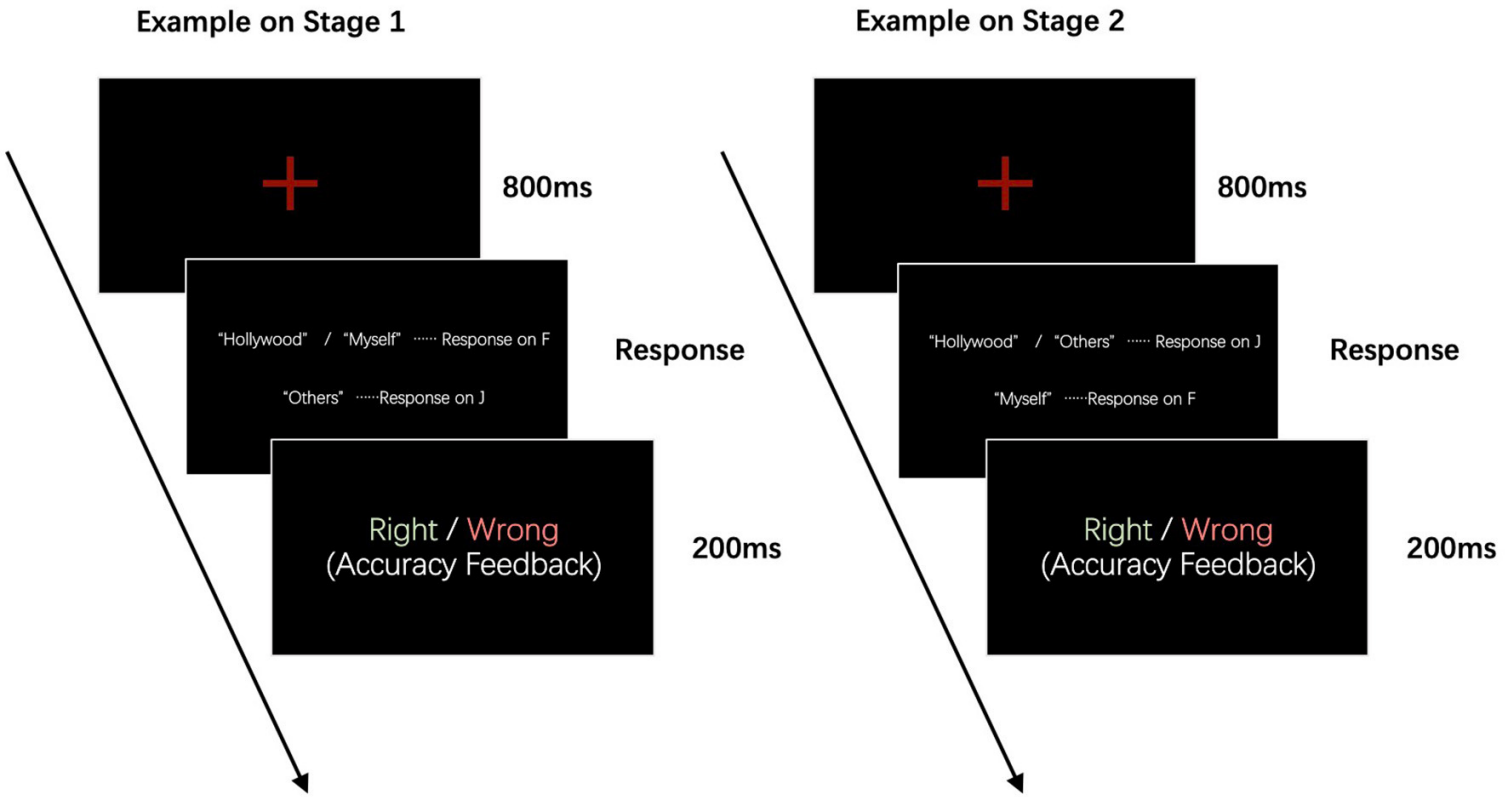
Examples of the procedure of SC-IAT. In stage 1, words describing “Western culture” and “self” were categorized on the “F” key, and words describing “others” were categorized on the “J” key; in stage 2, words describing “Western culture” and “others” were categorized on the J key, and words describing “self” were categorized on the F key.

The SC-IAT of intercultural awareness consisted of two stages summarized in Figure 2. Each stage included 24 exercises and 72 formal tests (24 tests in one round, a total of three rounds). Instructions were given concerning the categorization task and the appropriate key responses at the beginning. Words describing “Western culture” and “self” were categorized using the F key, and words describing “others” were categorized with the J key in stage 1. To prevent response bias, words describing “Western culture,” “self,” and “others” were presented in a ratio of 7:7:10. Thus, 58% of correct responses were with the F key, and 42% of correct responses used the J key. In contrast, words describing “Western culture” and “others” were categorized using the J key, and words describing “self” were categorized with the F key on the keypad in stage 2, with a similar procedure to that described above. The counterbalancing method was used to reduce the experimental error caused by sequence, fatigue, and practice effects. Figure 2 depicts examples of the experimental procedure.

Data quality control was first conducted before statistical analysis. Reaction times (RT) < 350 ms or more than 10,000 ms were eliminated, and the RT of error responses was replaced with the RT of stage mean plus an error penalty of 400 ms. D scores, the symbol of implicit effect, were calculated from the difference between the two stages' mean RT and then divided by the standard deviation of RT for all correct reactions (excluding the original wrong reactions) (52).


$$\begin{array}{l} \text{Implicit effect}:\text{ D = }({\text{Mean}}_{\text{reactions in stage1}}\\ {\text{ - Mean}}_{\text{reactions in stage2}}){\text{/Std}}_{\text{correct reactions}} \end{array}$$

Students with lower D scores tended to identify with Western culture.

#### Intercultural sensitivity

Based on the definition of intercultural sensitivity, Chen and Starosta (54) developed the Intercultural Sensitivity Scale (ISS). Huang revised the Chinese version of the scale in 2017, with a Cronbach's α coefficient of 0.865 indicating good reliability. The Cronbach's α in this study was 0.882. The Chinese version of the scale contains 20 items and four factors, which include openness, empathy, interaction confidence, and respect for cultural differences. The factor of openness refers to openness to the norms and values of different cultures. The empathy factor includes the ability to understand others' cognition and feelings in the intercultural context. Interaction confidence is individual confidence in intercultural communication. Respect for cultural differences refers to respect for the habits, customs, and behavior of individuals from different cultures (55). Options were on a 5-point Likert scale with the total score indicating the ability to develop positive emotions in an intercultural environment.

### Statistical analysis

Data were analyzed with SPSS version 21.0 and SPSSAU. The internal consistency coefficient of SC-IAT required *t*-tests for verification. *T*-tests were also used to verify differences in gender, D scores, and ISS scores between the two groups, which were classified using critical values for the SDS and SAS. ANOVA was used to compare age differences. Relationships between variables were examined by correlation analysis. Multivariate regression analysis was used to assess the possible predictors of anxiety and depression. We also set up a series of mediation models to investigate the associations between the possible predictors of anxiety and depression using a causal step approach.

The causal steps approach is an indirect test of the coefficient product for mediation (56). The following regression equation is used to describe the relationship between an independent variable *X*, a dependent variable *Y*, and a mediator variable *M*.


(1) $$\begin{array}{l} Y=cX+e_{1} \end{array}$$

(2) $$\begin{array}{l} M=aX+e_{2} \end{array}$$

(3) $$\begin{array}{l} Y=c^{\prime }X+bM+e_{3} \end{array}$$

The causal steps approach examines the total effect of *X* on *Y* in the first step by *c* in Equation (1) (i.e., inspection H0: *c* = 0). Then, the significance of the coefficient product is examined in step two by testing the coefficient a of Equation (2) (i.e., inspection H0: *a* = 0) and the coefficient b of Equation (3) (i.e., inspection H0: *b* = 0). The significance of coefficient a in Equation (2) indicates the significance of the effect from *X* to *M*. The significance of coefficient b in Equation (3) means that, when controlling the effect from *X*, the effect from *M* to *Y* is significant. The significance of coefficient c′ in Equation (3) means, when controlling the effect from *M*, the effect from *X* to *Y* is significant (direct effect). If coefficient c′ is significant in step one and coefficients a and b are significant in step two, the mediating effect is significant (56). If coefficient c′ is significant, the mediating effect is partial. Simulation studies have found that the rate of type-I error is low (57).

### Results

The Cronbach's α of SC-IAT was 0.80 in the present study—consistent with the range (0.55–0.85) of the internal consistency coefficient of SC-IAT in previous studies (52). Thus, the current version of SC-IAT should be reliable in its estimation of implicit intercultural identification.

With 53 points as the critical value of the SDS (*Mean* = 44.59, *SD* = 10.09) and 50 points as the critical value of the SAS (*Mean* = 40.50, *SD* = 9.92), eight students were considered to have apparent depression, while six students were thought to have apparent anxiety. We classified students into two groups based on whether their scores on the SDS/SAS were higher than the critical values. Group differences in D scores and scores of ISS were compared. The results indicated that D scores and the openness factor of ISS were significantly different between students with apparent anxiety or depression and students without anxiety or depression. In addition, total scores and the interaction confidence factor of ISS showed significant differences between students with apparent depression and students without apparent depression. The ISS respect for cultural difference factor also showed a significant difference between students with apparent anxiety and students without anxiety. No gender or age differences were found. Table 2 presents the differences in D score and the score of ISS classified on the critical value of SDS/SAS.

**Table 2:** Differences in D score and the score of ISS classified on the critical value of SDS/SAS (*N* = 34).

**Emotional disturbance**	**Variables**	**>53 (*****N*** = **8)**		≤ **53 (*****N*** = **26)**		***t***
		***M***	***SD***	***M***	***SD***	
SDS	D score	0.29	0.27	−0.02	0.27	2.74^**^
	ISS	74.88	8.25	82.85	7.26	−2.63^*^
	Openness of ISS	3.68	0.47	4.18	0.46	−2.70^*^
	Empathy of ISS	4.03	0.43	4.20	0.43	−1.01
	Interaction confidence of ISS	3.16	0.63	3.70	0.56	−2.36^*^
	Respect for cultural difference of ISS	4.25	0.46	4.55	0.40	−1.79
**Emotional Disturbance**	**Variables**	**>50 (N**= **6)**		≤ **50(N**= **28)**		***t***
		***M***	***SD***	***M***	***SD***	
SAS	D score	0.32	0.21	−0.00	0.29	2.57^*^
	ISS	75.83	7.00	82.07	8.04	−1.76
	Openness of ISS	3.67	0.42	4.14	0.48	−2.21^*^
	Empathy of ISS	4.03	0.32	4.19	0.45	−0.79
	Interaction confidence of ISS	3.46	0.58	3.60	0.62	−0.50
	Respect for cultural difference of ISS	4.17	0.41	4.55	0.41	−2.06^*^

M, means; SD, standard deviation; SDS, Self-rating Depression Scale; SAS, Self-rating Anxiety Scale; ISS, Intercultural Sensitivity Scale; ^*^p < 0.05; ^**^p < 0.01.

Table 3 presents descriptive statistics and correlations of all study variables. As expected, D scores showed a significant positive correlation with the SDS and SAS. The total ISS score and its subfactors—except for empathy—were negatively correlated with the SDS and SAS. Empathy only correlated negatively with the SAS. In addition, D scores only showed negative correlations with the openness of ISS. When the SDS and SAS were regressed on their related variables, respectively, D scores (β = 0.30, *p* = 0.022) and openness of ISS (β = −0.61, *p* < 0.001) could significantly predict SDS scores. SAS scores could also be predicted by D scores (β = 0.32, *p* = 0.030) and openness of ISS (β = −0.50, *p* = 0.001).

**Table 3:** Correlations among variables, means, and standard deviations (*N* = 34).

		***M***	***SD***	**Pearson** ***r***							
				**1**	**2**	**3**	**4**	**5**	**6**	**7**	**8**
1	SDS	44.59	10.09	–							
2	SAS	40.50	9.92	0.82^**^	–						
3	D score	0.05	0.30	0.51^**^	0.49^**^	–					
4	ISS	80.97	8.13	−0.64^**^	−0.57^**^	−0.31	–				
5	Openness of ISS	4.06	0.50	−0.71^**^	−0.60^**^	−0.34^*^	0.93^**^	–			
6	Empathy of ISS	4.16	0.43	−0.33	−0.36^*^	−0.29	0.77^**^	0.60^**^	–		
7	Interaction confidence of ISS	3.57	0.61	−0.49^**^	−0.37^*^	−0.11	0.76^**^	0.62^**^	0.38^*^	-	
8	Respect for cultural difference of ISS	4.48	0.43	−0.39^*^	−0.44^**^	−0.17	0.71^**^	0.60^**^	0.63^**^	0.31	-

M, means; SD, standard deviation; ISS, Intercultural Sensitivity Scale; ^*^p < 0.05; ^**^p < 0.01.

**Figure 3:**
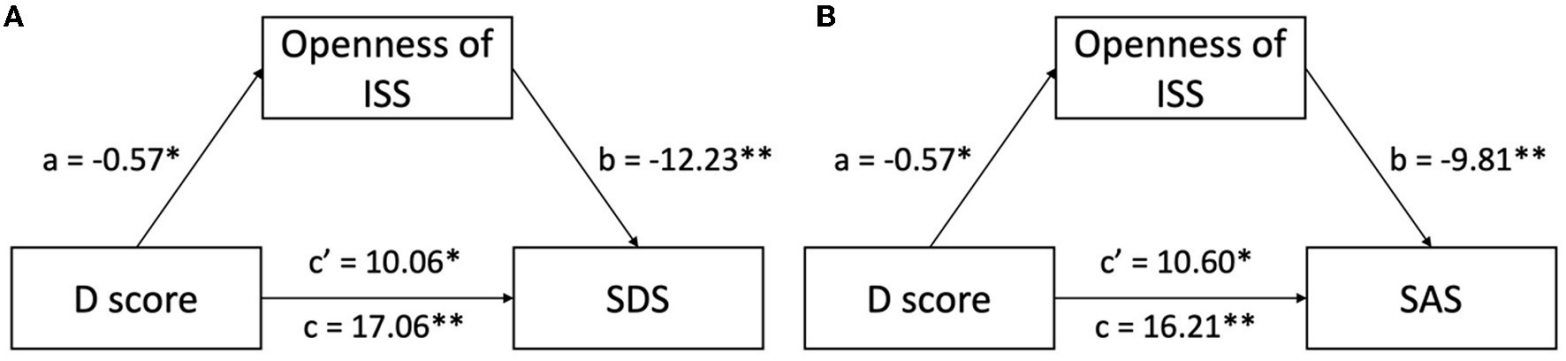
Openness factor mediates the linkage of implicit intercultural identification and emotional disturbances. **(A)** Openness of ISS partially mediates the association between implicit intercultural identification and depression levels; **(B)** openness of ISS partially mediates the association between implicit intercultural identification and anxiety levels. ISS, Intercultural Sensitivity Scale; SDS, Self-rating Depression Scale; SAS, Self-rating Anxiety Scale. ^*^p < 0.05, ^**^p < 0.01.

We established a series of mediation models to investigate the associations among the possible predictor (D score), mediator (ISS and its factors), and outcome variables (SDS and SAS). Table 4 shows the significant results of the mediation analysis. Model 1 revealed the effect of the D score on SDS, mediated by the openness factor of ISS. Model 2 revealed the effect of the D score on SAS, mediated by the openness factor of ISS. The total effect of the D score on SDS/SAS (*p* < 0.01) was significant, and the same results revealed the effect of the D score on the openness factor (*p* < 0.05) for both models. When controlling the D score, the effect of the openness factor on SDS/SAS was significant (*p* < 0.01). The effect of the D score on SDS/SAS was significant too (*p* < 0.05) when controlling the openness factor. The association between the D score and SDS (*c*′ = 10.06, *p* < 0.05, Figure 3A) and SAS (*c*′ = 10.60, *p* < 0.05, Figure 3B) was partially mediated through the openness factor of ISS. To estimate the size of the mediation effect, we calculated the ratio of the indirect to the total effect, as suggested by Wen and Ye (58). No other mediation models were found.

**Table 4:** Mediating effects on openness of ISS between D score and SDS/SAS.

**Model**	**Step**	**Dependent variable**	**Independent variable**	***B***	***R^2^***	***SE***	***t***
**1**	1 (Step c)	SDS	D score	17.06	0.26	5.12	3.33^**^
	2 (Step a)	Openness	D score	−0.57	0.12	0.28	−2.07^*^
	3 (Step b) (Step c')	SDS	Openness	−12.23	0.58	2.50	−4.88^**^
			D score	10.06		4.17	2.41^*^16.5pt
	**Indirect effect**	**Direct effect**	**The ratio of indirect effect to the total effect**				
	7.00	10.06	41.04%				
	**Step**	**Dependent variable**	**Independent variable**	***B***	***R** ^2^*	***SE***	***t***
2	1 (Step c)	SAS	D score	16.21	0.24	5.09	3.19^**^
	2 (Step a)	Openness	D score	−0.57	0.12	0.28	−2.07^*^
	3 (Step b) (Step c')	SAS	Openness	−9.81	0.46	2.80	−3.50^**^
			D score	10.60		4.67	2.27^*^
	**Indirect effect**	**Direct effect**	**The ratio of indirect effect to the total effect**				
	5.61	10.60	34.65%				

SDS, Self-rating Depression Scale; SAS, Self-rating Anxiety Scale; ISS, Intercultural Sensitivity Scale. ^*^p < 0.05; ^**^p < 0.01.

### Discussion

Study 2 presents a complex picture of the relationship between intercultural communication competence and emotional disturbance. As Hypothesis 2 stated, an emotional disturbance is closely related to different aspects of intercultural communication competence. The openness and interaction confidence of intercultural sensitivity in students with apparent depression were significantly different from those without depression. Intercultural sensitivity was also significantly different between apparently anxious and non-anxious students, especially for the factors of openness and respect for cultural differences. This is consistent with prior studies which suggested that students with lower levels of intercultural sensitivity experience more anxiety and depression (14, 42). Emotions and feelings are unstable in adolescence (44), and students with higher levels of intercultural sensitivity might be less affected by adolescent emotional instability in the intercultural context than students with lower levels of intercultural sensitivity.

Students who do not implicitly identify with the new culture experienced more emotional distress, according to a meta-analysis of 38 studies indicating a statistically significant negative relationship between intercultural identification and depression (59). Intercultural conflicts might lead to identification confusion in adolescents attending international high schools, which could result in a more challenging process of intercultural adaptation and more emotional disturbance (30, 31). Furthermore, we found that both openness to intercultural sensitivity and implicit intercultural identification with the new culture could predict students' stages of anxiety and depression. Meanwhile, the effect of implicit intercultural identification on emotional disturbance was partially mediated by the openness factor of intercultural sensitivity, with 41.04% of indirect effect to total effect on depression and 34.65% on anxiety. Students with higher implicit intercultural identification with the new culture had greater open-mindedness in the affective aspect regarding their experiences in the new environment, which could help them move away from negative emotions—just partially, as Hypothesis 3 stated. This is consistent with studies that found that openness is closely connected to intercultural attitudes conducive for intercultural adaptation (39, 40).

## General discussion

The current study aimed to elucidate the prevalence of emotional disturbances, such as depression and anxiety, and their relationship with key competences of intercultural adaptation in new students in international high schools. Study 1 investigated the prevalence of depression and anxiety among senior-one students. Previous studies pointed out that intercultural adaptation might play an important role in mental health. Thus, Study 2 further explored whether intercultural communication competence protects against emotional disturbance. We also examined how implicit intercultural identification and intercultural sensitivity—representing cognitive and affective aspects of intercultural communication competence—work during the process of intercultural adaptation.

Our results indicate that several students in their 1st year of international school are affected by negative emotions. Some students might not have demonstrated symptoms of anxiety or depression following admission because of protective factors related to initial curiosity about the new context—as was the case for research on Afghan students at universities in Malaysia where most students were excited at the beginning of their intercultural journey (60). Another qualitative study focusing on intercultural communication also suggested that intercultural curiosity is associated with a positive experience (61). Nevertheless, theories of intercultural adaptation indicate that curiosity will not last long—only 2 weeks to 2 months. After the initial period of idealization, individuals often experience cultural shock and emotional difficulties (4). Kim (62, 63) suggested that intercultural adaptation is a process of intercultural identity acquisition through a stress–adaptation–growth cycle involving a full spectrum of emotions including joy, despair, and anger. After the idealization phase, individuals may be troubled by difficulties in the new intercultural environment and experience severe stress until they have satisfactorily adapted. Adaptation is a circular process of continuous progress. The psychological impact may disturb individuals, but it also promotes active adjustment to intercultural contexts. Therefore, the progress and adjustment of international high school students in senior grades should be further explored.

Kim's stress-adaptation-growth dynamic theory (63) suggests that the ability to communicate in accordance with the norms and practices of an intercultural context is central to intercultural adaptation. Two interrelated experiences are involved in the process of intercultural adaptation. Deculturation involves the loss of one's heritage culture, while acculturation refers to the acquisition of a new culture. The process is described as “a subtle and largely unconscious shift from monocultural to an increasingly intercultural self-other orientation,” ultimately leading to intercultural identity transformation (63). Previous studies confirmed this process and suggested that individuals demonstrating intercultural integration adapt more readily to the intercultural environment and have better mental health (64, 65). The literature is consistent with our results that students who were reluctant to identify with the new culture were more likely to be depressed and anxious. Individuals who refuse to identify with the new culture lack cognitive regulation that can mitigate against conflicts between themselves and the intercultural environment. The vicious circle of a lack of identification, mental health challenges, and conflict with the environment may induce further depression (66). Disidentification means that individuals in a new culture may be unwilling to acquire intercultural knowledge proactively. They will face more uncertainty in intercultural communication, which leads to social anxiety (14, 67).

Students in our research were in an international high school in East Asia with a western cultural context. Thus, their negative emotions during the process of intercultural adaptation might correlate with the conflict between intercultural identification at the implicit level of intercultural awareness and the external culture. Previous research with Korean–American young adults indicated that explicit and implicit intercultural identification differed for individuals in intercultural environments. This inconsistency could be explained by the expression of a contradictory attitude of the socially underrepresented group to social identity, related to psychological pain (68). Few studies have concentrated on students' intercultural adaptation and intercultural identification in international high schools located in areas with obvious intercultural conflicts. Our preliminary research found a negative relationship between implicit intercultural identification and emotional disturbances, but it remains unclear how implicit intercultural identification impacts emotional adjustment mechanisms during intercultural adaptation. This field deserves further attention.

While intercultural sensitivity, the affective aspect of intercultural communication competence, also relates closely to emotional disturbance (42), our results indicated that senior-one students with lower levels of intercultural sensitivity suffered from more anxiety and depression. Chen (4) attributed this to emotional resistance when facing a new culture. Individuals experience both curiosity and uneasiness at the outset of their intercultural journey. Emotional resistance can help to temporarily isolate oneself from feelings of uneasiness and feel better (62). However, such a response might engender a further refusal to adapt, leading to delayed emotional distress. The affective aspect of intercultural communication competence refers to empathy for the new culture and attachment to others within it and may help students adapt to a new culture with less uneasiness and resistance.

Additionally, the effect of implicit intercultural identification on emotional disturbances was partially mediated by the openness factor of intercultural sensitivity. This finding from our study corresponds to the suggestion that the affective aspect is based on the cognitive aspect of intercultural communication competence (38, 69). Openness refers to the willingness of individuals to openly and appropriately explain their own culture, accept the differences and needs of other cultures, and transform such emotions into actions (38). Openness can help individuals manage the psychological threat of the intercultural environment to obtain more creative benefits from intercultural integration (70). Openness is also related to wellbeing determinants consisting of emotional feelings (71).

Thus, international schools should pay more attention to students' mental health during intercultural adaptation. Teachers need to understand the process of culture shock and impart relevant knowledge to their students to reduce students' confusion when experiencing a new culture and struggling with negative emotions. Moreover, psychological counselors in international schools should keep a watchful eye on the emotional symptoms associated with intercultural adaptation. Compared with students from other countries, fewer Asian students seek help from mental health services (72). Thus, it is essential to take a proactive approach to offer psychological support in Asian countries. This support could help students overcome the difficulties associated with cultural shock in international high schools and have a positive impact on students' future lives in foreign counties. Previous studies also suggested that setting up a course to improve the intercultural communication competence of college students studying abroad is imperative (73). Our results indicate that the cultivation of intercultural communication competence is also critical for students in international schools.

## Strengths and limitations

The field of intercultural adaptation has received much attention alongside the promotion of cultural globalization. Research over the past 3 decades has provided a basis for the establishment of theoretical models of intercultural adaptation, and the focus of intercultural research has narrowed into more specific strands. The process of intercultural adaptation may be similar for all populations, but unique, population-specific aspects may also exist. Only by exploring across research studies, we can learn the characteristics of the intercultural adaptation process in different groups more specifically.

A unique aspect of the present study was our research object. Globalization will inevitably promote the demand for intercultural talent and an increasing desire for international education. Individuals who need to adapt to interculture will become younger. Data show that the number of high school students in international schools is increasing, but research on the process of intercultural adaptation in this group is scarce. Understanding the process of intercultural adaptation for students in international high schools will help them maintain their mental health in an intercultural environment and can further promote the exploration of intercultural adaptation in younger groups.

Moreover, most previous studies have focused on evaluating cultural traits and differences—the lower levels of intercultural awareness. We used SC-IAT to evaluate higher levels of intercultural awareness, conducive to the formation of autonomy for individuals to appreciate cultural differences and enjoy intercultural life. Our results represent participants' implicit cognition, which is more stable than explicit cognition derived from questionnaires and less affected by social expectations.

However, several limitations should be acknowledged. Previous research with foreign students in universities has indicated that the same cultural context has different effects on the process of intercultural adaptation of individuals from different regions (74). As an exploratory study, cluster sampling was used with our participants in an international high school to avoid the impact of regional differences on results. Further surveys in international high schools in other regions are needed to determine whether there are any differences between students from different areas. Sample sizes should also be increased. The present study was a cross-sectional survey, which did not allow for causal explanations. We plan to test our hypotheses longitudinally using a long-term survey in the future to advance our understanding of student characteristics at different stages of international high school education.

## Conclusion

Our results revealed that many students in their 1st year of international school are affected by depression and anxiety. Their emotional disturbances significantly correlated with intercultural sensitivity and implicit intercultural identification, representing affective and cognitive aspects of intercultural communication competence, respectively. The openness factor of intercultural sensitivity played a mediating role between implicit intercultural identification and emotional disturbance. The intercultural communication competence of students in their 1st year of international high school needs to be promoted to protect them from mental health difficulties.

## Data availability statement

The raw data supporting the conclusions of this article will be made available by the authors, without undue reservation.

## Ethics statement

The studies involving human participants were reviewed and approved by Institutional Review Board (IRB) of The Department of Psychology, Southern Medical University. Informed consent was provided by the participants and their legal guardian/next of kin. Informed consent in Study 1 collected by Internet while it was collected by written in Study 2. Informed consent was obtained from the individual(s), and minor(s)' legal guardian/next of kin, for the publication of any potentially identifiable images or data included in this article.

## Author contributions

RZ and JH conceived and designed this study. JH conducted statistical analyses and drafted the manuscript, with reference to additional advice provided by RZ, XS, and CW. All authors edited and approved the final manuscript.
